# Effects of weight loss and insulin reduction on arterial stiffness in the SAVE trial

**DOI:** 10.1186/1475-2840-11-114

**Published:** 2012-09-22

**Authors:** Timothy M Hughes, Andrew D Althouse, Nancy A Niemczyk, Marquis S Hawkins, Allison L Kuipers, Kim Sutton-Tyrrell

**Affiliations:** 1 Department of Epidemiology, Graduate School of Public Health, University of Pittsburgh, 130 N. Bellefield Street, Room 463, Pittsburgh, PA, USA

**Keywords:** Pulse wave velocity, Insulin, Weight loss and arterial stiffness

## Abstract

**Background:**

Chronic arterial stiffness contributes to the negative health effects of obesity and insulin resistance, which include hypertension, stroke, and increased cardiovascular and all-cause mortality. Weight loss and improved insulin sensitivity are individually associated with improved central arterial stiffness; however, their combined effects on arterial stiffness are poorly understood. The purpose of this study was to determine how insulin levels modify the improvements in arterial stiffness seen with weight loss in overweight and obese young adults.

**Methods:**

To assess the effects of weight loss and decreased fasting insulin on vascular stiffness, we studied 339 participants in the Slow the Adverse Effects of Vascular Aging (SAVE) trial. At study entry, the participants were aged 20–45, normotensive, non-diabetic, and had a body-mass index of 25–39.9 kg/m^2^. Measures of pulse wave velocity (PWV) in the central (carotid-femoral (*cf*PWV)), peripheral (femoral-ankle (*fa*PWV)), and mixed (brachial-ankle (*ba*PWV)) vascular beds were collected at baseline and 6 months. The effects of 6-month change in weight and insulin on measures of PWV were estimated using multivariate regression.

**Results:**

After adjustment for baseline risk factors and change in systolic blood pressure, 6-month weight loss and 6-month change in fasting insulin independently predicted improvement in *ba*PWV but not *fa*PWV or *cf*PWV. There was a significant interaction between 6-month weight change and change in fasting insulin when predicting changes in *ba*PWV (p < 0.001). Individuals experiencing both weight loss and insulin reductions showed the greatest improvement in *ba*PWV.

**Conclusions:**

Young adults with excess weight who both lower their insulin levels and lose weight see the greatest improvement in vascular stiffness. This improvement in vascular stiffness with weight loss and insulin declines may occur throughout the vasculature and may not be limited to individual vascular beds.

**Trial registration:**

NCT00366990

## Background

Paramount among the negative health effects of obesity and insulin resistance (IR) are their adverse effects on vascular health [1]. Arterial stiffness is a measure of vascular health and its progression is accelerated in both obesity and IR [2,3]. Chronic arterial stiffness eventually disrupts the hemodynamics of the vasculature, contributing to hypertension, stroke, and both cardiovascular and all-cause mortality [4].

Pulse wave velocity (PWV) is a non-invasive measure of arterial stiffness. Higher values are associated with greater stiffness. Weight loss is associated with reductions in PWV, independent of baseline weight [5]. These findings were recently replicated in the Slow Adverse Vascular Effects (SAVE) clinical trial, preliminary results from which demonstrate that a weight loss and physical activity intervention can reduce arterial stiffness in young adults with excess weight [6]. Weight loss also directly reduces insulin levels and IR [7]. Emerging research indicates that obesity alone may not pose short term risk of cardiovascular events, and that IR may be a key factor in the obese individual’s increased risk for cardiovascular disease [8]. While weight loss [5] and increased insulin sensitivity [2] are individually associated with improved central arterial stiffness, their combined effects on the aorta and other arterial segments are poorly understood. Understanding the contributions of weight loss and improved insulin sensitivity to arterial stiffness will provide insight into sub-clinical vascular changes preceding and potentially contributing to complications of diabetes and hypertension.

Recent work from our group explored potential predictors of change in pulse-wave velocity and found that changes in BMI, heart rate and C-reactive protein were associated with changes in PWV [9]. However, these analyses did not assess the effects of changes in insulin and weight on PWV. The purpose of this analysis was to specifically examine the combined effects of reduction in both weight and insulin on changes in pulse wave velocity measured in various arterial locations in young overweight, sedentary adults engaged in a weight loss intervention. We hypothesized that: 1) weight loss and insulin reductions would independently be associated with reductions in pulse wave velocity measured across various arterial sites, and 2) the combined effect of weight loss and insulin reductions would be significantly related to improvements in pulse wave velocity.

## Methods

### SAVE population

This study consists of 339 healthy men and women who had viable measures of baseline PWV and additional information on covariates from the 349 subjects who participated in the SAVE clinical trial (NCT00366990). Methods of participant recruitment and intervention in the SAVE trial were previously reported [10]. Briefly, SAVE is a randomized controlled trial examining the effects of weight reduction, physical activity and dietary sodium reduction on improving vascular health. Participants were 25 – 45 year-old men and women from Allegheny County, PA, who were physically inactive and overweight to class II obese. Exclusion criteria for SAVE included: 1) diabetes, defined as fasting blood glucose ≥126 mg/dl; 2) hypertension, defined as average screening blood pressure ≥ 140/90 mmHg or any treatment with antihypertensive medications; 3) current use of cholesterol-lowering, antipsychotic, or vasoactive medicines or devices; 4) any underlying inflammatory condition; 5) known atherosclerotic disease; and 6) pregnancy, breastfeeding, or plans for pregnancy during the study period. All study participants signed an informed consent document approved by the University of Pittsburgh Institutional Review Board.

### Main outcomes

Pulse wave velocity was assessed at the University of Pittsburgh Ultrasound Research Laboratory (Pittsburgh, PA, US) using a noninvasive and automated waveform analyzer (Colin Co., Komaki, Japan) [11]. All measures were performed in a quiet temperature-controlled room after a 12 hour fast and abstinence from caffeine. Individuals were also asked to refrain from vigorous exercise thirty minutes prior to testing.

Resting blood pressure was measured twice after a 10 minute rest using an automated device. Blood pressure cuffs were attached to both uncovered arms and ankles using appropriate cuff sizes. Electrocardiogram clips were attached to both wrists. A phonocardiogram was held in place with a two pound weight at the fourth intercostal space to the left of the sternum, and tonometry sensors were manually placed on the left carotid and femoral arteries.

PWV was measured in the central (carotid-femoral (*cf*PWV), peripheral (femoral-ankle (*fa*PWV)), and mixed (brachial-ankle (*ba*PWV)) vascular beds. PWV is calculated as the distance in centimeters between arterial sites of interest over time (in seconds) that the pressure waveforms travel from the heart to the respective arterial sites. Distance for *cf*PWV was calculated by subtracting the distance from the carotid artery to suprasternal notch from the sum of the distances between suprasternal notch to the inferior edge of the umbilicus and from the inferior edge of the umbilicus to the femoral artery. Distances between peripheral sites were calculated using a height based algorithm as required by the Colin waveform analyzer [11]. Time was calculated using the foot-to-foot velocity method of waveforms measured at various sites [12]. The average of two runs was used in this analysis. The validity and reliability of pulse wave velocity assessment with this device has been previously reported [13]. Ultrasound Research Laboratory staff determined the reproducibility of PWV measures, using intraclass correlation coefficients (ICC). ICC was higher for *ba*PWV (ICC = 0.97) and *fa*PWV (ICC = 0.96) compared to *cf*PWV (ICC = 0.75).

### Main predictors

Insulin was measured using standard radio-immune assay (Linco Research, St. Charles, MO). Insulin change was calculated as the difference in insulin levels from baseline to 6 months follow-up. HOMA-IR, which is a measured of insulin resistance, was calculated as (glucose x insulin)/405 [14].

Research staff measured weight on the same standard balance beam scale at each visit. Participants were instructed to fast, empty their bladders and remove their shoes, jackets, and heavy sweaters before being weighed. Weights were rounded up to the nearest 1/10 of a kilogram. Weight change was the difference in weight from baseline to 6 months follow-up. BMI was calculated for each study visit as weight in kilograms divided by height in meters squared.

### Covariates

Self-reported information was collected from participants regarding age, gender, race, and smoking status. Staff measured height and blood pressure using standardized protocols. Blood pressures were measured using a conventional manual mercury sphygmomanometer. Staff measured the diameter of the midpoint of the right upper arm to determine correct cuff size. Participants rested for 5 minutes with uncrossed arms and feet flat on the floor, and were instructed not to speak during the rest period or blood pressure measurement. Readings were taken with participants sitting with their arm elevated, so that the blood pressure cuff was at heart level. After staff determined the maximum inflation level, they used the bell of the stethoscope to identify the first and fifth Korotkoff sounds. The latter 2 of 3 readings taken at least 30 seconds apart were averaged together. Mean arterial pressures (MAP) was calculated as:

(1) $$\text{MAP}=\left(1/3\right)*\text{averaged SBP}+\left(2/3\right)*\text{averaged DBP}$$

Laboratory assays were performed on fasting serum samples at the Heinz Laboratory at the University of Pittsburgh’s Graduate School of Public Health (Pittsburgh, PA, USA). Total cholesterol [15], high density lipoprotein (HDLc) [16], low density lipoprotein (LDLc) [17], and glucose were determined using standard laboratory procedures. C-reactive protein was measured with an enzyme-linked immunoassay (Alpha Diagnostics International, Inc. San Antonio, TX).

### Statistical methods

Variables with normal distributions are presented as mean ± SD; variables with skewed distributions are presented as median [inter-quartile range]. Longitudinal changes in PWV are reported as mean ± SD as well as percentage change from baseline measurement. T-tests were used to test for differences in longitudinal PWV by gender and baseline fasting insulin levels.

Since the primary aim of this analysis was to determine if participants with both weight loss and reduction in insulin over six months had a greater combined effect on changes in pulse-wave velocity than those without change in either, we performed a post-hoc power calculation and determined we had 80% power to detect at least a 38 cm/sec difference between the individuals with the combined effect weight loss and insulin reductions and those without.

Six month change in weight, insulin and PWV was calculated by subtracting the six month value from baseline values. Simple linear regression was used to estimate the univariate effects of change in weight and change in insulin on PWV in each arterial bed (Model 1). Multiple regression analysis was used to estimate the simultaneous effects of change in weight and insulin on change in PWV separately for each of the three PWV outcomes (Model 2). Potential covariates were then forced into the multiple regression model to adjust for baseline differences and changing in mean arterial pressure. After adjusting for baseline age, sex, race, smoking status, BMI, MAP, HDLc, triglycerides, CRP, fasting insulin, PWV and 6-month change in MAP, a multiple linear regression analysis was performed to identify any independent associations between weight loss, change in insulin level, and change in PWV. In order to assess the combined effect of weight loss and insulin reductions on changes in PWV, we added an interaction term to models where weight loss and insulin reductions were simultaneously associated with changes in PWV (Model 3). We reran the models of *cf*PWV after removing potential outliers (e.g. two individuals with change in *cf*PWV > 400 cm/sec) and determined that inclusion of outlying values had no effect on these models.

In a separate model, we adjusted for SAVE intervention assignment; however, the randomized treatment had no effect on the reported relationships and is not reported here. In sensitivity analyses, we reassessed each model of PWV with HOMA-IR change as a predictor in place of insulin change, and found insulin levels to be a stronger predictor of changes in PWV than HOMA-IR. All models presented in this paper report changes in insulin levels. Insulin is reported as the primary predictor because it explained more of the variance in PWV measures than HOMA-IR in the SAVE trial of individual with fasting glucose levels within the normal range. All statistical analyses were performed using SAS statistical software release 9.2 (SAS Institute Inc., Cary, NC).

## Results

Baseline characteristics for participants by gender are displayed in (Table 1). Participants had a mean age of 37.9 years (range 21–46 years) and ranged from overweight to class II obese (mean BMI 32.8 kg/m^2^) with a mean weight of 92.2 kg. The sample was predominantly female (77.0%), white (80.5%) and never smokers (62.2%). Age and BMI did not differ between genders. Men were also more likely to be white and never smokers than women (P = 0.013 and 0.067, respectively). Fasting glucose, mean SBP and mean diastolic blood pressure (DBP) were within normal ranges in the overall sample, consistent with study entry criteria. However, the men had significantly greater fasting glucose, SBP and DBP than women at baseline (P < 0.05 for all), even though they were within normal values.

**Table 1:** Baseline risk factors and vascular stiffness in the SAVE trial

	**All (N = 339)**		**Women (N = 261)**		**Men (N = 78)**		
***Risk Factor***	**Mean**	**SD**	**Mean**	**SD**	**Mean**	**SD**	**P-Value**^**‡**^
Age (years)	37.9	6.1	37.5	6.1	38.0	6.1	0.478
Weight (kg)	92.2	15.0	88.3	12.6	105.1	15.3	<0.001
BMI (kg/m^2^)	32.8	3.9	32.6	3.9	33.5	3.7	0.062
Heart Rate (bpm)	70.0	17.4	69.9	16.5	70.0	20.4	0.998
Total Cholesterol (mg/dL)	203.3	37.4	203.5	38.0	202.7	35.7	0.876
HDLc (mg/dL)	52.8	13.5	55.8	13.4	42.7	7.9	<.001
LDLc (mg/dL)	123.7	33.2	122.7	33.9	126.9	30.4	0.329
Triglycerides (mg/dL)^†^	116	(78–170)	106	(76–155)	151	(110–209)	<0.001
CRP (mg/dL)^†^	2.6	(1.3-5.7)	2.9	(1.5-6.5)	2.2	(0.9-3.6)	<0.001
Fasting Insulin (μU/mL)	14.6	8.1	14.0	6.8	16.5	11.2	0.069
HOMA-IR	3.6	2.1	3.4	1.8	4.1	2.9	0.045
Systolic BP, mm Hg	113.3	10.5	112.2	10.6	117.1	9.1	<.001
Diastolic BP, mm Hg	72.9	8.7	71.8	8.6	76.5	8.1	<.001
Mean Arterial Pressure	93.1	8.9	92.0	8.9	96.8	7.9	<.001
Ankle-Brachial Index	1.06	0.07	1.05	0.07	1.08	0.07	0.002
Race – n,%							0.013
*White*	273	80.5%	205	78.5%	68	87.2%	
*Black*	54	15.9%	49	18.8%	5	6.4%	
*Other*	12	3.5%	7	2.7%	5	6.4%	
Smoking Status – n,%							0.067
*Never*	211	62.2%	155	59.4%	56	71.8%	
*Former*	96	28.4%	82	31.4%	14	17.9%	
*Current*	32	9.4%	24	9.2%	8	10.3%	
**Vascular Stiffness**	**Mean**	**SD**	**Mean**	**SD**	**Mean**	**SD**	
Mixed, *ba*PWV (cm/sec)	1207.6	132.3	1194.2	126.0	1252.3	143.6	<.001
Peripheral, *fa*PWV (cm/sec)	945.9	102.8	942.9	98.2	956.1	117.5	0.385
Central, *cf*PWV (cm/sec)	880.0	257.4	864.6	247.8	932.3	283.1	0.046

^*^ Values reported are mean ± SD unless otherwise noted.

^†^ Reported as Median (Q1-Q3) due to skewed distribution.

^‡^ Denotes test for difference between men and women.

HOMA-IR: Homeostatic Insulin Resistance, CRP: C - reactive protein; *ba*PWV: brachial-ankle pulse-wave velocity (n = 339); *fa*PWV: femoral-ankle pulse-wave velocity (n = 320); *cf*PWV: carotid-femoral pulse-wave velocity (n = 324).

Other cardiovascular risk factors were not as optimal as glucose and blood pressure Table 1. Despite being a non-diabetic cohort, participants had elevated baseline insulin levels and HOMA-IR. Total cholesterol, LDLc and C-reactive protein (CRP) were also at the high end of the normal ranges. Men recruited into SAVE tended to have a more adverse cardiometabolic risk profile than women in SAVE, with significantly higher glucose, HOMA-IR, SBP, DBP, MAP and triglycerides and lower HDLc than women. Only CRP was more adverse in women. Men had greater baseline PWV than women in all arterial beds except for *fa*PWV. Brachial-ankle PWV differed most between genders at baseline (48 cm/s higher in men on average compared to women, P < 0.001). The measures of baseline arterial stiffness were significantly correlated with one another (baPWV and faPWV, r = 0.69, p = <0.001; baPWV and cfPWV, r = 0.30, p = <0.001; faPWV and cfPWV, r = 0.10, p = 0.084).

Both men and women lost weight and decreased their insulin levels over the 6 month intervention. Men lost an average of 9.3 kg of weight and 2.5 μU/mL of insulin compared with an average loss of 5.8 kg and 1.0 μU/mL for women. Participants had significant decreases in *ba*PWV and *cf*PWV but not in *fa*PWV over the 6-month period (Table 2). Similar trends were seen in the men alone; however, in women, only *cf*PWV decreased significantly. The difference by gender was only significant for *ba*PWV, which decreased by 33.3 cm/s in men and 4.1 cm/s in women (P = 0.045).

**Table 2:** Six-month changes in vascular stiffness and risk factors during the SAVE trial

	**SAVE (N = 272)**		
***Vascular Stiffness***	**Mean**	**SD**	**% Change**
Δ *ba*PWV (cm/s), mixed	−11.6†	91.5	−0.7
Δ *fa*PWV (cm/s), peripheral	+5.8	91.9	+0.9
Δ *cf*PWV (cm/s), central	−51.9†	303.3	−1.6
***Potential Covariates***			
Weight (kg)	−7.0	5.9	−7.6
BMI (kg/m^2^)	−2.0	2.0	−7.0
HDLc (mg/dL)	0.9	8.0	3.1
LDLc (mg/dL)	−2.9	25.7	−0.3
Triglycerides (mg/dL)	−18.7	60.1	−9.1
CRP (mg/dL)	−1.0	4.9	−38.0
Fasting Insulin (μU/mL)	−1.3	6.9	−0.7
Systolic BP, mm Hg	−2.6	8.2	−2.0
Diastolic BP, mm Hg	−1.5	7.7	−1.4
Mean Arterial Pressure	−2.0	7.2	−1.9

* Values reported represent the crude mean change from baseline PWV and the % change from baseline PWV.

† change from zero, p < 0.05.

*ba*PWV: brachial-ankle pulse-wave velocity.

*fa*PWV: femoral-ankle pulse-wave velocity.

*cf*PWV: carotid-femoral pulse-wave velocity.

**Figure 1:**
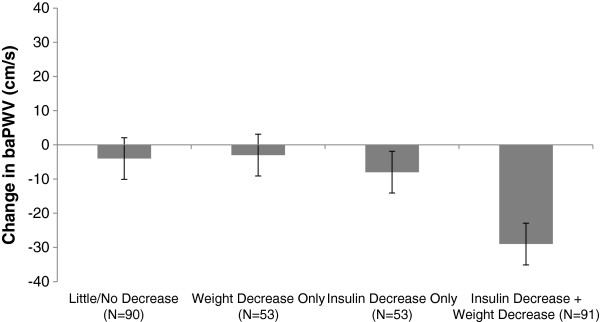
Adjusted^* ^**mean changes in *ba*PWV by insulin- and weight-loss groups in the SAVE trial.** Baseline and 6-month mean PWV measures are plotted by weight and insulin-loss groups where groups are split by median changes in weight and fasting insulin levels. ^*^Adjusted for age, sex, race, smoking status and baseline BMI, mean arterial pressure, HDLc, triglycerides, CRP, fasting insulin, *ba*PWV and change in mean arterial pressure. *ba*PWV: brachial-ankle pulse-wave velocity.

Insulin reduction and weight loss were individually associated with decreases in *ba*PWV (p = 0.023 and p = 0.040, respectively, Table 3), but not with *fa*PWV or *cf*PWV (data not shown). Individually, weight loss and insulin reductions predicted decreases in *ba*PWV independent of baseline cardiometabolic risk factors, including: age, sex, race, smoking status, BMI, SBP, HDLc, triglycerides, CRP, fasting insulin, and baseline PWV (p = 0.083 and 0.097, respectively). Subjects with elevated baseline insulin (fasting insulin ≥15μU/mL) experienced a mean decrease of 19.2 cm/sec in *ba*PWV, compared to an increase of 0.1 cm/sec in patient with normal baseline insulin. After adjustment for insulin change, weight loss was no longer significantly associated with decreases in *ba*PWV (p = 0.0681). There was a significant interaction effect of insulin change and weight change (Table 3, P < 0.001) for changes in *ba*PWV independent of other cardiovascular risk factors. These results for absolute changes in insulin and weight were consistent with models where weight and insulin change were expressed as a percent change from baseline measures. Participants with both weight loss and insulin reductions had the greatest improvement in *ba*PWV (Figure 1). The PWV data showed that individuals in the upper tertile of the *ba*PWV distribution experienced greater than a 50 cm/s decrease in *ba*PWV over six months. Reaching this benefit was strongly predicted by both insulin change and weight loss. Additional file 1: Figure S1 shows the incremental benefit of weight loss and changes in insulin on *ba*PWV. The proportion of individuals reaching a 50 cm/s declines in *ba*PWV was similar for those experiencing little/no change as those with weight loss only (28% versus 26%, respectively). This levels of benefit was achieved by 35% of participants with insulin reduction only, and by 45% of those with both reduced weight and insulin.

**Table 3:** Linear regression analyses predicting six-month changes in brachial-ankle pulse-wave velocity in the SAVE trial

**Predictor**	**Unadjusted Associations**^**1**^		**Model 2**^**2**^		**Model 3**^**3**^	
	**β**	**95% CI**	**β**	**95% CI**	**β**	**95% CI**
Insulin Reduction	−1.71**	(−0.08, -3.34)	−0.83	(0.77, -2.43)	3.71	(2.52, 5.00)
Weight Loss	−2.14**	(−0.32, -3.98)	−1.55*	(0.57, -3.61)	−0.95	(−1.92 ,0.02)
Interaction	--	--	--	--	−0.43***	(−0.31, -0.55)

*P < 0.10, **P < 0.05, ***P < 0.001.

^1^ In the unadjusted model, the **β** term represents the predicted change in pulse-wave velocity (cm/s) per unit **decrease** in insulin (μU/mL) and weight (kg).

^2^ In the second column of multivariate models, the **β** term represents the predicted change in pulse-wave velocity (cm/s) per unit **decrease** in insulin (μU/mL) and weight (kg) when adjusting for age, sex, race, smoking status, mean arterial pressure, HDL cholesterol, triglycerides, CRP, baseline *ba*PWV and change in mean arterial pressure. This is one model with both weight loss and insulin change simultaneously plus covariates.

^3^ In the third column of multivariate models, the **β** term represents the predicted change in pulse-wave velocity (cm/s) for a **simultaneous decrease** in insulin (μU/mL) and weight (kg) when adjusting for age, sex, race, smoking status, mean arterial pressure, HDL cholesterol, triglycerides, CRP, baseline *ba*PWV and change in mean arterial pressure. This is one model with both weight loss and insulin change simultaneously plus their interaction plus covariates.

*ba*PWV: brachial-ankle pulse-wave velocity.

## Discussion

This work demonstrates that both weight loss and insulin reductions had direct and positive effects on arterial stiffness, and that the strongest effect occured in individuals who simultaneously lost weight and lowered their insulin levels. Specifically, weight loss and insulin reductions were individually associated with improvements in *ba*PWV independent of cardiometabolic risk factors. The combined effect of weight loss and insulin reduction over 6-months had a greater impact on *ba*PWV than weight loss or insulin reductions alone.

Weight loss has been previously shown to improve vascular stiffness [9,15] although the mechanism underlying this association is unknown. Previous work from the SAVE trial has studied various predictors of changes in pulse-wave velocity [9]. The current analysis was directed at studying the relationship between weight loss, improved insulin sensitivity, and subsequent changes in vascular stiffness. We found that the combined effects of weight loss and reduction in circulating insulin was independent of changes in other factors related to change in pulse-wave velocity [9]. Weight loss affects a wide variety of physiologic measures, including insulin sensitivity and circulating insulin levels, in part, through improved insulin homeostasis [18]. Hyperinsulinemia promotes the arteriosclerotic process by instigating vascular smooth muscle cells proliferation and migration in the vessel wall [18,22]. Recently Yue et al., in a study of people with type 2 diabetes, found that poor glycemic control was associated with lower circulating levels of endothelial progenitor cells and higher *ba*PWV [23]. PWV is also associated with other measures of arterial architecture, including vascular thickness [9,24]. Results from the ongoing Vaso Risk study [25] have the potential to provide more detail on the relationship between diabetes, glycemic control and arterial stiffness.

The observed results differ by location within the arterial tree. Weight loss and insulin reduction were associated with reductions in *ba*PWV, a mixed measure of stiffness, but not with changes in purely central or peripheral measures. These data suggest improvements in weight and insulin are likely to be systemic and can be seen in measures of the vasculature that incorporate both central and peripheral PWV. While central measures of PWV changed in response to the lifestyle intervention in SAVE, weight loss and insulin reductions were not associated with changes in these measures. The individual and combined effects of weight loss and insulin reductions were more pronounced in the mixed measure than the purely central and peripheral measures. Our findings suggest that using mixed measures of PWV that take into account both central and peripheral vascular changes is the best representation of the effects of weight and insulin change on arterial stiffness. These findings add to previous studies that show weight loss is associated with improvements in both central [5,26] and mixed [9] measures of arterial stiffness. The effects of weight loss and insulin declines are more apparent throughout the vasculature than in central and peripheral arterial beds alone.

It is possible that we only detected significant differences in *ba*PWV because this mixed measure of arterial stiffness is more sensitive to short-term vascular remodeling. However, it is also possible that we did not see effects in *cf*PWV as a result of its large variances at baseline and follow up, resulting in limited statistical power to detect small changes over time. The addition of carotid and femoral probes makes the PWV protocol more difficult to execute and may result in more error in the peripheral measures especially in obese individuals [27]. In previous analysis, our group identified factors other than changes in insulin as potential predictors of cfPWV [9]. Furthermore, the removal of potential outliers (thirty individuals with change in *cf*PWV >400 cm/sec) had little effect on the results of these analyses and weight loss and insulin reduction still did not predict changes *cf*PWV.

In this analysis, adjustment for insulin change fully attenuated the association between weight loss and decreases in *ba*PWV. This evidence builds on the findings of previous studies that show insulin levels and impaired fasting glucose are more related to peripheral measures of PWV than BMI, weight and weight loss. A study of 697 non-diabetic adults found increased *ba*PWV in subjects with impaired fasting glucose, independent of BMI [28]. While these results imply that there is a connection between impaired fasting glucose and mixed measures of arterial stiffness, their study did not measure fasting insulin levels and could not conclude that the effect of impaired glucose levels on *ba*PWV were independent of insulin levels [28]. Zhang et al., found that Chinese people with type 2 diabetes had increased central and peripheral PWV compared to controls, and fasting glucose was a determinant of peripheral artery stiffness. This study did not measure fasting insulin levels or relate these levels to neither fasting glucose levels or arterial stiffness [29]. Recently, Rudofsky et al. reported the effects of 12 weeks of weight loss intervention in 21 obese and non-diabetic participants on PWV, measured by finger photoplethysmography [15]. They showed that improvements in PWV were associated with improvements in HOMA-IR only, and not with weight loss.

The effect of weight loss and insulin reduction on *ba*PWV was stronger in men. The differential effect of sex is likely explained by differences in the baseline characteristics of men and women recruited for the SAVE trial. At SAVE baseline, men had greater risk factors for CVD and diabetes and higher baseline PWV than women. As a result, the SAVE intervention had a greater impact on weight loss and cardiometabolic risk factors in men. In the first six months of the SAVE trial, men lost more weight than women (9.3 kg vs. 5.8 kg) and had more than twice the decrease in insulin (2.5 μU/mL vs. 1.0 μU/mL). Our findings suggest that the weight loss intervention was more successful in men than women, as has been reported previously in the literature [5,30].

The relatively small numbers of men and African-Americans in the SAVE trail limits our ability to generalize to these groups and to stratify all analyses by gender or race, although adjustment for these factors was made. Additionally our study only focused on the 6-month change in weight, insulin and PWV. Consistent with other findings, the SAVE study found that weight loss plateaus between 6–12 months of follow up; consequently, we limited our analysis to 6-month data to avoid instances of weight re-gain in study subjects.

The SAVE trial provides longitudinal assessment of arterial stiffness and cardiometabolic risk factors. While the participants in SAVE were overweight or obese, they were generally healthy, without impaired fasting glucose or diabetes [8]. The SAVE trial focuses on this segment of the population enabling the study of the effects of obesity and the early stages of insulin resistance prior to the onset of diabetes. Obese individuals with hyperinsulinemia are potentially ideal targets for such an intervention targeted to lower PWV. Our study shows that in terms of cardiovascular risk, people with hyperinsulinemia and obesity will likely show the most benefit from a weight loss intervention. Further, the measurement of regional PWV provides additional insight into the correlates of arterial stiffness along the vasculature, which a single assessment could not provide. Future studies of weight loss and changes in arterial stiffness should utilize additional measures of metabolic syndrome [31] and diabetes risk factors [32] to elucidate the relationships between weight loss, diabetes risk factors and arterial stiffness.

## Conclusions

Young overweight and obese adults who lower their insulin levels and lose weight over 6 months experience decreased vascular stiffness, measured by PWV. This improvement in vascular stiffness was greatest among individuals who experienced both weight loss and insulin reduction. The potential effects of these reductions on arterial stiffness may be more pronounced throughout the vasculature than in the individual vascular beds. Overweight and obese individuals with elevated insulin levels are likely to have the greatest improvement in arterial stiffness from clinical interventions focused on weight loss and decreased blood insulin levels.

## Competing interests

The authors have no competing interests to report. Timothy M. Hughes, Andrew D. Althouse, Marquis Hawkins and Allison Kuipers were supported by an NHBLI training grant to the University of Pittsburgh (T32HL083825). Nancy A. Niemczyk was supported by an NICHD training grant to the University of Pittsburgh (T32HD0055162-04). Kim Sutton-Tyrrell is the principle investigator of SAVE, supported by NHBI (R01 HL077525-01A2).

## Authors’ contributions

Timothy Hughes and Andrew Althouse were responsible for the design, data analysis and drafting of the manuscript. Marquis Hawkins, Allison Kuipers, Nancy A. Niemczyk and Kim Sutton-Tyrrell contributed to the drafting and editing of the manuscript. Kim Sutton-Tyrrell was the primary investigator of the SAVE trial (NCT00366990). All authors read and approved the final manuscript.

## Supplementary Material

Additional file 1 **Figure S1.** Proportion of subjects experiencing a decrease in baPWV of ≥50 cm/sec by insulin- and weight-loss groups in the SAVE trial. This more clearly shows that a greater proportion of the “Weight Decrease and Insulin Decrease” had a baPWV decrease of 50 cm/sec (*Note: I chose 50 as somewhat of an arbitrary cutoff, but it seems to provide a decent # of patients in all categories and also will appeal to readers that just like round numbers). There is also a statistically significant difference between the groups in a global test (p = 0.015), so it might be appealing to present this figure with a p-value. Click here for file
