# Supplementary material for: Effects of weight loss and insulin reduction on arterial stiffness in the SAVE trial
Source: Cardiovasc Diabetol. 2012 Sep 22;11:114. doi: 10.1186/1475-2840-11-114 (PMC3468408; doi:10.1186/1475-2840-11-114)
Supplement: Additional file 1 — Figure S1. Proportion of subjects experiencing a decrease in baPWV of ≥50 cm/sec by insulin- and weight-loss groups in the SAVE trial. This more clearly shows that a greater proportion of the “Weight Decrease and Insulin Decrease” had a baPWV decrease of 50 cm/sec (*Note: I chose 50 as somewhat of an arbitrary cutoff, but it seems to provide a decent # of patients in all categories and also will appeal to readers that just like round numbers). There is also a statistically significant difference between the groups in a global test (p = 0.015), so it might be appealing to present this figure with a p-value. [file 1475-2840-11-114-S1.doc]

Supplemental Figure 1. **Proportion of subjects experiencing a decrease in baPWV of ≥50 cm/sec by insulin- and weight-loss groups in the SAVE trial**


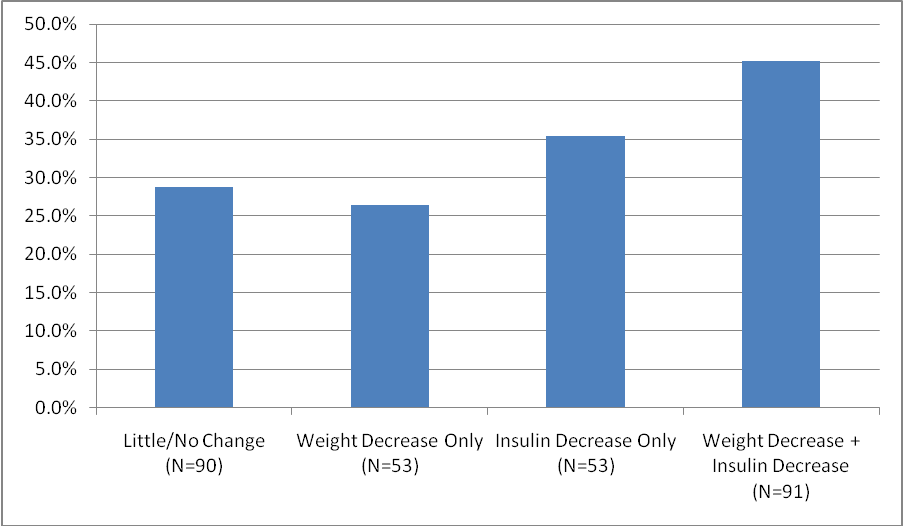


This more clearly shows that a greater proportion of the “Weight Decrease and Insulin Decrease” had a baPWV decrease of 50 cm/sec (*Note: I chose 50 as somewhat of an arbitrary cutoff, but it seems to provide a decent # of patients in all categories and also will appeal to readers that just like round numbers). There is also a statistically significant difference between the groups in a global test (p=0.015), so it might be appealing to present this figure with a p-value.
